# Pulsed field ablation for paroxysmal supraventricular tachycardia: a systematic review and single-arm meta-analysis

**DOI:** 10.1007/s10840-025-02166-w

**Published:** 2025-11-21

**Authors:** Giang Son Arrighini, Mrunalini Dandamudi, Vinh Quang Tri Ho, David Downes, Nicole Felix, Vanio L J Antunes, Guilherme Dagostin de Carvalho, Iuri Ferreira Felix, Victoria Zecchin Ferrara, Hemank Walia, Igor Diemberger, Gabriel Odozynski, Andre d’Avila, Juliana Giorgi

**Affiliations:** 1 Faculty of Medicine and Surgery, University of Bologna, Bologna, Italy; 2 Montefiore Einstein Hospital, Bronx, NY USA; 3 Faculty of Medicine, University of Debrecen, Debrecen, Hungary; 4 Department of Rural Medicine, The University of New England, Armidale, NSW Australia; 5 Federal University of Campina Grande, Campina Grande, PB Brazil; 6 Federal University of Health Sciences of Porto Alegre, Porto Alegre, RS Brazil; 7 Department of Cardiac Arrhythmias and Electrophysiology, Dante Pazzanese Institute of Cardiology, São Paulo, Brazil; 8 Department of Medicine, Mayo Clinic, Rochester, MN USA; 9 Faculty of Medicine and Surgery, University of Padua, Padua, Italy; 10 Advocate Illinois Masonic Medical Center, Chicago, IL USA; 11 Department of Cardio-Thoraco-Vascular Sciences, Cardiology Unit, IRCCS Azienda Ospedaliero-Universitaria Di Bologna, Bologna, Italy; 12 Harvard-Thorndike Electrophysiology Institute, Beth Israel Deaconess Medical Center, Harvard Medical School, Boston, MA USA; 13 Hospital Sírio-Libanês, São Paulo, SP Brazil; 14 Hospital Albert Einstein, São Paulo, SP Brazil

**Keywords:** Pulsed field ablation, Paroxysmal supraventricular tachycardia, Atrioventricular nodal reentrant tachycardia, Atrioventricular reentrant tachycardia, Accessory pathways, Meta-analysis

## Abstract

**Background:**

Pulsed field ablation (PFA) is a novel, non-thermal technique for atrial fibrillation ablation that is currently under early investigation for paroxysmal supraventricular tachycardia (PSVT). We conducted a meta-analysis to evaluate the efficacy and safety of PFA in this setting.

**Methods:**

We systematically searched PubMed, Embase, Cochrane Central, and Web of Science for studies on PFA in PSVT, including atrioventricular nodal reentrant tachycardia (AVNRT) and atrioventricular reentrant tachycardia (AVRT) with concealed or manifest accessory pathways. Outcomes included acute ablation success, success at 1, 3, and 6 months, and procedural/postoperative adverse events (AEs). Proportions were pooled using a random-effects model with arcsine transformation to account for extreme values. Analyses were conducted using R (v4.3.2).

**Results:**

Five prospective single-arm studies involving 202 patients were included. The pooled acute success rate was 99.98% (95% CI: 99.29–100), with sustained success at 1, 3, and 6 months. Procedural and postoperative AE rates were low: 0.92% (95% CI: 0.00–4.88) and 0.06% (95% CI: 0.00–0.87), respectively. Subgroup analysis showed 100% acute success in AVNRT and in AVRT with concealed pathways, with high sustained success at 6 months. In AVRT with manifest pathways, acute success was 97.5% (95% CI: 84.74–100), maintained through follow-up.

**Conclusion:**

In this systematic review and meta-analysis, PFA demonstrated excellent safety and efficacy for the treatment of PSVT. Randomized controlled trials are warranted to establish the outcomes of PFA in this setting relative to thermal ablation.

**Graphical Abstract:**

**Figure:**
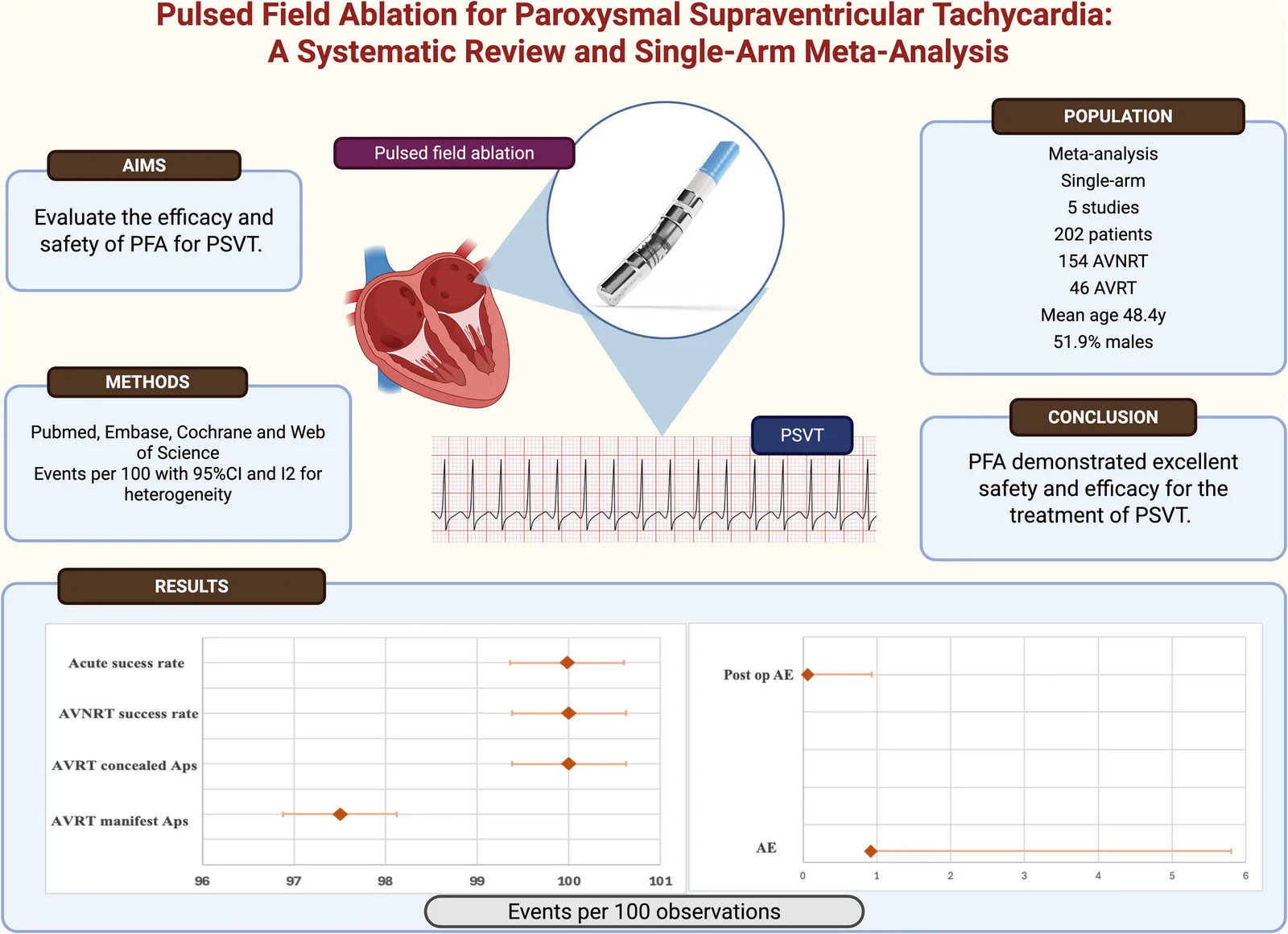

**Supplementary Information:**

The online version contains supplementary material available at https://doi.org/10.1007/s10840-025-02166-w.

## Introduction

Pulsed field ablation (PFA) has emerged as a promising alternative to traditional thermal ablation techniques, such as radiofrequency ablation (RFA) and cryoablation, for the treatment of persistent and paroxysmal atrial fibrillation [1]. Unlike thermal methods, PFA utilizes ultrafast electric fields to induce irreversible electroporation, resulting in selective cell death primarily in myocardial tissue. This mechanism offers the potential to minimize damage to non-target structures, such as the His bundle, which could theoretically make PFA a safer approach for managing paroxysmal supraventricular tachycardia (PSVT) [2, 3].

However, current evidence supporting PFA remains limited, primarily derived from single-center studies with small sample sizes. Consequently, there are uncertainties surrounding its broader applicability and cost-effectiveness. This systematic review and single-arm meta-analysis seeks to address these gaps by synthesizing data from recent studies to evaluate the safety and feasibility of PFA for PSVT. By pooling data from smaller cohorts, this analysis aims to provide a comprehensive assessment of current knowledge, identify potential subgroups that may benefit most, and inform the design of future multicenter trials. To our knowledge, this is the first systematic review and meta-analysis on this topic.

## Methods

This systematic review and meta-analysis were conducted and reported in accordance with the Cochrane Collaboration Handbook for Systematic Reviews of Interventions and the Preferred Reporting Items for Systematic Reviews and Meta-Analysis (PRISMA) Statement guidelines [4, 5]. As such, the prospective protocol was registered with the International Prospective Register of Systematic Reviews (PROSPERO) under protocol number CRD42024621050.

### Eligibility criteria

Inclusion in this meta-analysis was restricted to studies that met all the following criteria: (1) patients aged 18 or older; (2) diagnosed with PSVT; (3) who underwent PFA; (4) and had at least one clinical outcome of interest.

We excluded studies with (1) overlapping populations; (2) case report or case series design; (3) conference abstracts; (4) and enrolling only patients with atrial fibrillation, atrial flutter or atrial tachycardia.

### Search strategy

We systematically searched PubMed, Embase, Cochrane Central, and Web of Science to identify eligible studies. The search was conducted from inception to November 30, 2024, using the following strategy: [("pulsed field ablation" OR PFA OR electroporation) AND ("paroxysmal supraventricular tachycardia" OR PSVT OR "supraventricular tachycardia" OR SVT OR "atrioventricular nodal reentrant tachycardia" OR AVNRT OR "atrioventricular reentrant tachycardia" OR “atrioventricular reentry tachycardia” OR AVRT OR "nodal reentrant tachycardia" OR "Wolff-Parkinson-White syndrome" OR WPW OR "accessory pathway" OR “accessory pathways”)].

No filters, language restrictions, or publication date limits were applied to the search. Additionally, we performed a backward snowballing search by manually reviewing the reference lists of included studies and relevant review articles.

Two authors (G.S.A. & M.D.) independently evaluated study titles and abstracts using predefined search parameters to select studies that met the inclusion criteria. Then, the same two authors assessed the complete texts for inclusion and gathered data. Disagreements were resolved by consensus. G.S.A and V.H.Q.T. subsequently examined all selected articles and collected the data following predefined criteria.

### Endpoints and subgroup analyses

Our primary outcomes of interest included the acute ablation success rate, as well as success rates at 1, 3, and 6 months post-procedure. Success was defined based on specific electrophysiological endpoints.

For atrioventricular nodal reentrant tachycardia (AVNRT), successful ablation was confirmed by the elimination of dual AV node physiology and the inability to induce tachycardia through programmed stimulation. The persistence of slow pathway conduction was considered acceptable only if it was non-inducible [6, 7].

For atrioventricular reentrant tachycardia (AVRT), success was defined by the complete elimination of conduction across the accessory pathway, evidenced by the absence of preexcitation on surface electrocardiogram and the inability to induce tachycardia during programmed stimulation. Post-ablation, adenosine testing was used to confirm the absence of dormant conduction in the accessory pathway. Additionally, isoproterenol infusion during programmed atrial stimulation was employed to enhance adrenergic tone, which could uncover latent conduction or arrhythmia susceptibility, ensuring comprehensive validation of procedural success and reducing the risk of recurrence [8].

Ablation success rates at 1, 3, and 6 months were defined as the absence of recurrent, sustained PSVT, confirmed by 12-lead electrocardiogram or 24-h Holter monitoring, without the use of antiarrhythmic drugs. One study outlined follow-up visits at 1–3 months, with additional check-ups planned at 6–12 months; however, it did not specify whether all patients were evaluated at exact time points, such as 6 months. For our analysis, we included data from this study in the time periods closest to our study's follow-up intervals [9].

Safety outcomes of interest included procedural and postoperative adverse events (AEs), specifically: atrioventricular block (AVB), intraventricular block, ventricular arrhythmia, myocardial infarction, hemolysis or major bleeding requiring transfusion, cardiac tamponade or perforation, pericarditis requiring treatment, infectious endocarditis, deterioration of tricuspid valve stenosis or regurgitation, acute heart failure, abnormal renal or hepatic function, intracerebral hemorrhage, transient ischemic attack, stroke, and all-cause mortality.

Additional outcomes included procedural characteristics, such as mean total ablation time, average skin-to-skin procedure time, average fluoroscopy time, average contact force, and the mean distance from the closest ablation site to the His electrogram cloud tagged in the mapping system or the tagged His signal.

A subgroup analysis was conducted for outcomes of interest, focusing on AVNRT, AVRT with concealed accessory pathways (APs), and AVRT with manifest APs.

In addition, we extracted for each study the follow-up duration, the monitoring modality used to assess success (e.g., 12-lead ECG, Holter, event recorder), and the number of patients lost to follow-up.

### Quality assessment

Two authors (D.D. and I.F.) assessed risk of bias using the “Risk of Bias in Non-randomized Studies – of Interventions” (ROBINS-I) tool [10]. Disagreements were resolved through a consensus after discussing the reasons for the discrepancy.

### Statistical analysis

We conducted a single-arm meta-analysis to estimate the pooled incidence of outcomes across the included studies. The proportion for each study was calculated as the number of events divided by the total number of participants, with results expressed as percentages and 95% confidence intervals (CIs). For continuous outcomes, the pooled mean and standard deviation were used for the synthesis.

The Der Simonian-Laird random-effects model, along with the inverse variance method and arcsine transformation, was applied to calculate the pooled proportions. This approach was chosen to address convergence issues and to account for studies where the proportion was 100% (i.e., 1), which often caused the study variance to approach zero, resulting in disproportionately large, hidden study weights. Unlike logit or logarithmic transformations, this method uses inverse trigonometric (arcsine) functions to stabilize the variance of proportions across studies. Continuous outcomes were pooled with the same settings except for the absence of transformations.

Additionally, confidence intervals (CIs) for proportions were estimated using the Clopper-Pearson method as the default in the *meta* package. Heterogeneity among studies was assessed using the I^2^ statistic. Forest plots were created to visually display the pooled proportions and continuous outcomes along with study-specific estimates. A test for subgroup differences with a p value < 0.05 was considered statistically significant for different subgroup results. All statistical analyses were performed using R version 4.3.2.

### Assessment of publication bias

Due to the limited number of included studies, we did not perform formal publication bias assessment using funnel plots or Egger’s test, as these methods have low power and reliability when fewer than 10 studies are available. To mitigate potential publication bias, we conducted a comprehensive literature search without language or date restrictions and manually reviewed trial registries and reference lists.

### Sensitivity analysis

We conducted a leave-one-out sensitivity analysis for all outcomes to assess the potential impact of individual studies on the overall results. Additionally, we performed a sensitivity analysis using the Freeman-Tukey double arcsine transformation to evaluate the robustness of our statistical approach.

## Results

### Study selection and baseline characteristics

**Fig. 1:**
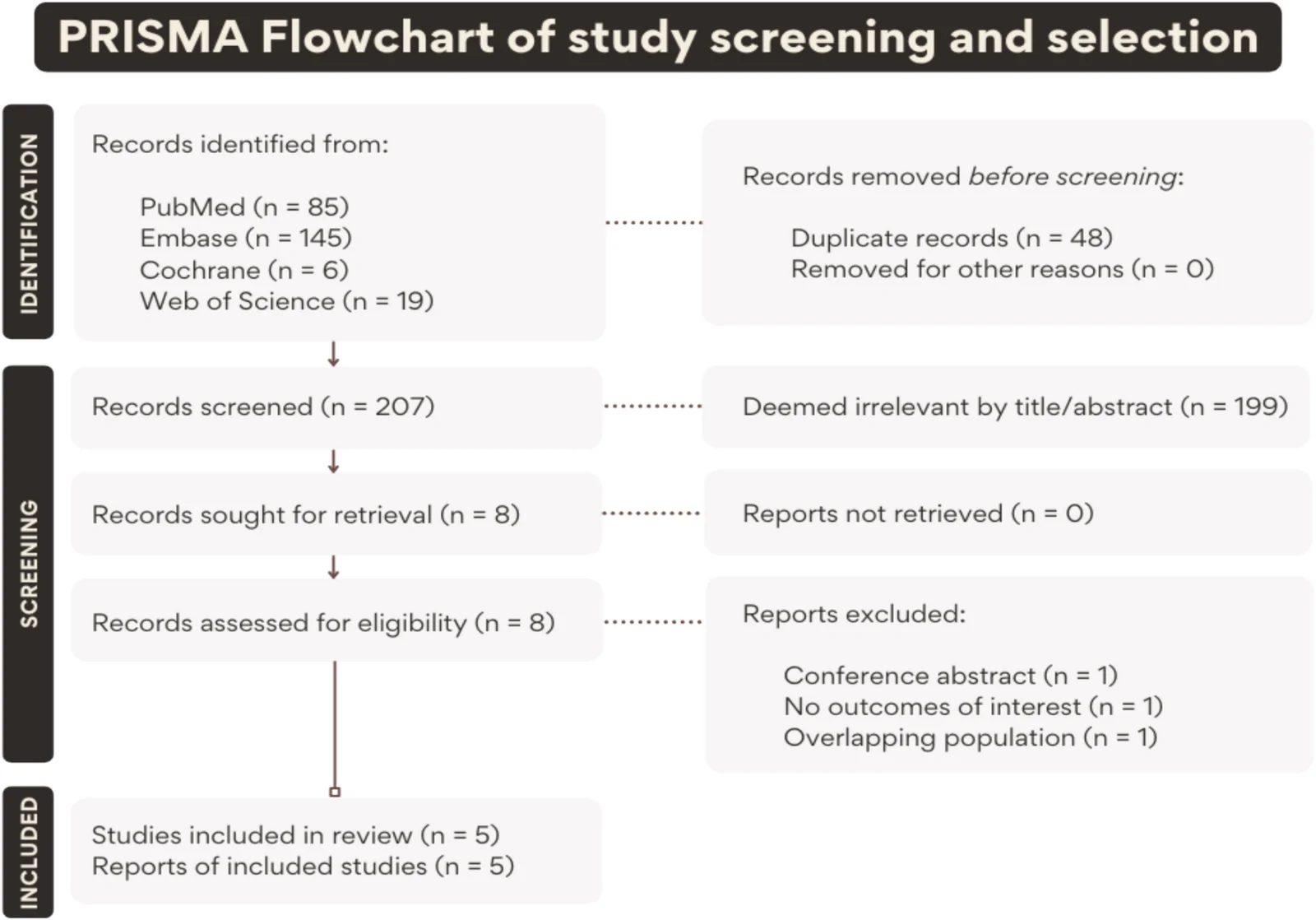
PRISMA flow diagram of study screening and selection

The initial search yielded 255 results. After removing duplicates and excluding ineligible studies, eight studies were thoroughly reviewed by the inclusion criteria. Of these, five non-randomized trials were included [2, 3, 9, 11, 12], comprising 202 patients (Fig. 1).

A total of 202 patients underwent PFA for PSVT. Study characteristics are summarized in Table 1. Of these patients, 154 had AVNRT, 27 had AVRT with concealed APs, 19 had AVRT with manifest APs, and two had both AVNRT and AVRT. Four studies were prospective, single-arm, single-center trials [2, 9, 11, 12], while one study was a prospective, multicenter, single-arm trial [3]. The mean age across the studies was approximately 48.4 years, and the median percentage of male participants was 51.9%. One study included three pediatric patients, who were excluded from this analysis [9]. Additionally, another study included patients who underwent both PFA and RFA, and these patients were also excluded from the analysis [3] In this study, RFA was not employed as a rescue strategy after failed PFA; rather, it was performed as a planned consolidation step exclusively for patients with AVRT and far-His accessory pathways (> 10 mm from the His bundle) after acute procedural success with PFA had already been achieved, as per the predefined study protocol. Details regarding follow-up duration, monitoring modalities, and loss to follow-up are reported in Table 2.

**Table 1:** Baseline characteristics of included studies

Study	Design	Study period	N. of patients	Male, n (%)	Age,y, mean ± SD	BMI, kg/m2	PSVT duration, months	AVNRT	AVRT with concealed APs	AVRT with manifest APs	AVNRT and AVRT
Breskovic 2024	Single-center, Single-arm	December 2022 to January 2024	11	8 (72.73)	37.5 ± 13				2	9	
Li 2024 †	Multicenter, Single-arm	January 2023 to August 2023	158 (111 PFA only)	82 (51.9)	44.7 ± 14.6	23.96 ± 4.40		77 (PFA only)	63 (23 PFA only)	16 (9 PFA only)	2 (PFA only)
Shen 2024 (a)	Single-center, Single-arm	October 2022 to January 2023	10	6 (60)	46.8 ± 14.4	22.8 ± 3.01	66.4 ± 82.9	7	2	1	
Shen 2024 (b)	Single-center, Single-arm	March 2023 to November 2023	40	13 (32.5)	52.2 ± 15.9	23.8 ± 3.4	51.1 ± 93.9	40			
Zeng 2024	Single-center, Single-arm	May 2022 to November 2022	30	12 (40.0)	47.9 ± 13.9	24.0	2.0 (2.0- 4.0)	30			

*AP*, Accessory Pathway; *AVNRT*, Atrioventricular Nodal Reentrant Tachycardia; *AVRT*, Atrioventricular Reentrant Tachycardia; *BMI*, Body Mass Index; *PSVT*, Paroxysmal Supraventricular Tachycardia; † Li et al. included the entire population, combining PFA and RFA, due to the lack of baseline characteristics specific to PFA alone

**Table 2:** Follow-up duration, monitoring modality, loss to follow-up of included studies

Study	Follow-up duration	Monitoring modality	Loss to follow-up
Breskovic et al	1, 3, and 6 months	12-lead ECG at 1–3 months, 24-h Holter at 6–12 months, 2 with ILR	No mention of loss to follow-up
Li et al	14 days, 3 months, 6 months	12-lead ECG (14d), 24-h Holter (90d & 180 d), transesophageal pacing (AVNRT/AVRT)	No mention of loss to follow-up
Shen et al. (a)	1, 3, and 6 months	12-lead ECG & 24-h Holter at 1, 3, 6 months; EPS at 1 and 6 months	No mention of loss to follow-up
Shen et al. (b)	1, 3, and 6 months	12-lead ECG & 24-h Holter at 1, 3, 6 months; echo and blood test at 1 month; EPS optional	No mention of loss to follow-up
Zeng et al	14 days, 3 months, 6 months	24-h Holter at each visit; echo & blood tests at 14 days and 3 months	No mention of loss to follow-up

*ECG*, Electrocardiogram; *EPS*, Electrophysiological study; *Holter*, 24-h Holter monitoring; *ILR*, Implantable loop recorder; *Echo*, Transthoracic echocardiography; *AVNRT*, Atrioventricular nodal reentrant tachycardia; *AVRT*, Atrioventricular reentrant tachycardia; *d*, days

### Procedural characteristics

Due to the use of new technology and varying procedural setups, the procedural details have been summarized in Supplementary Material (Table S1) for clarity and ease of reference.

### Pooled analysis of all studies

**Fig. 2:**
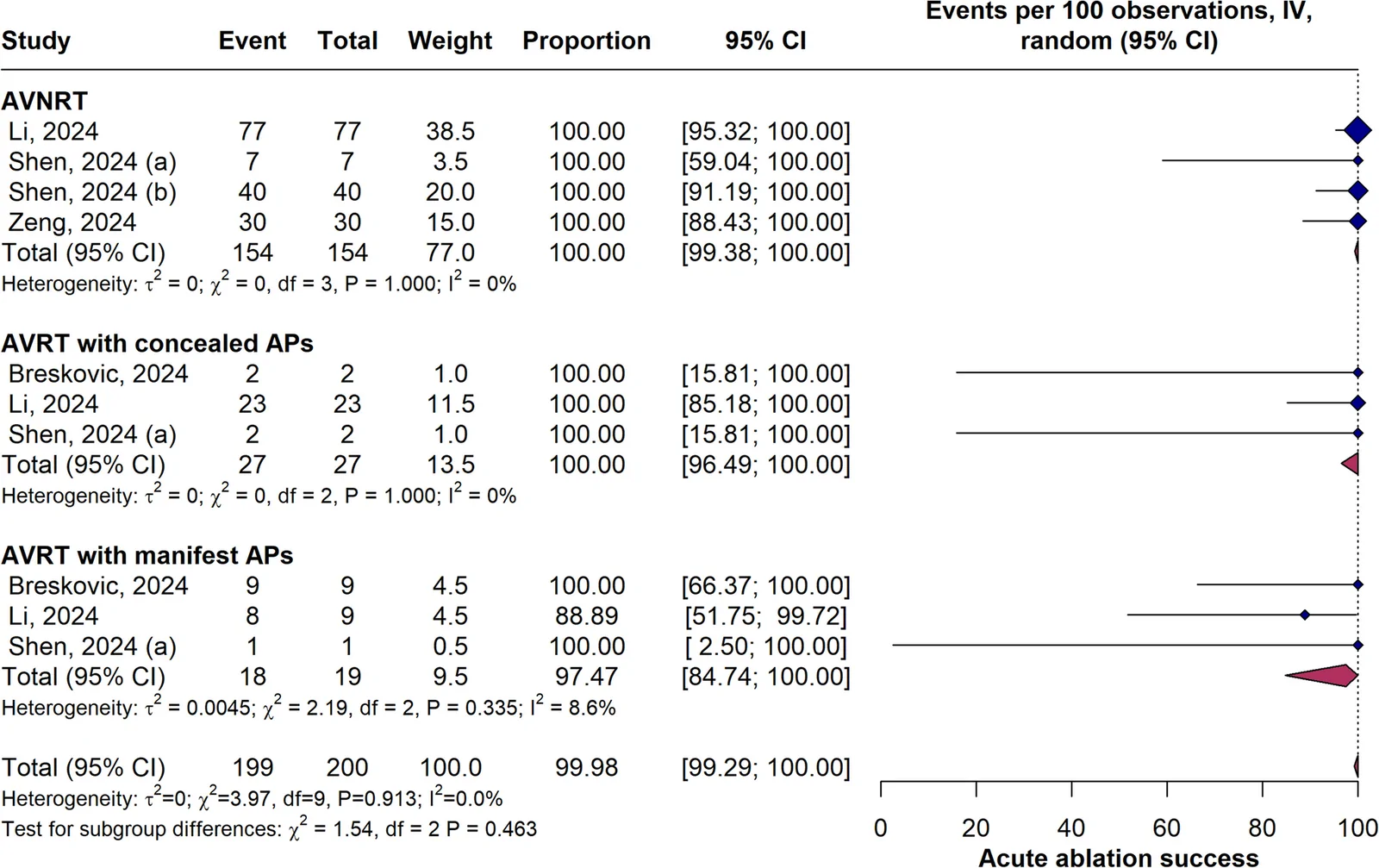

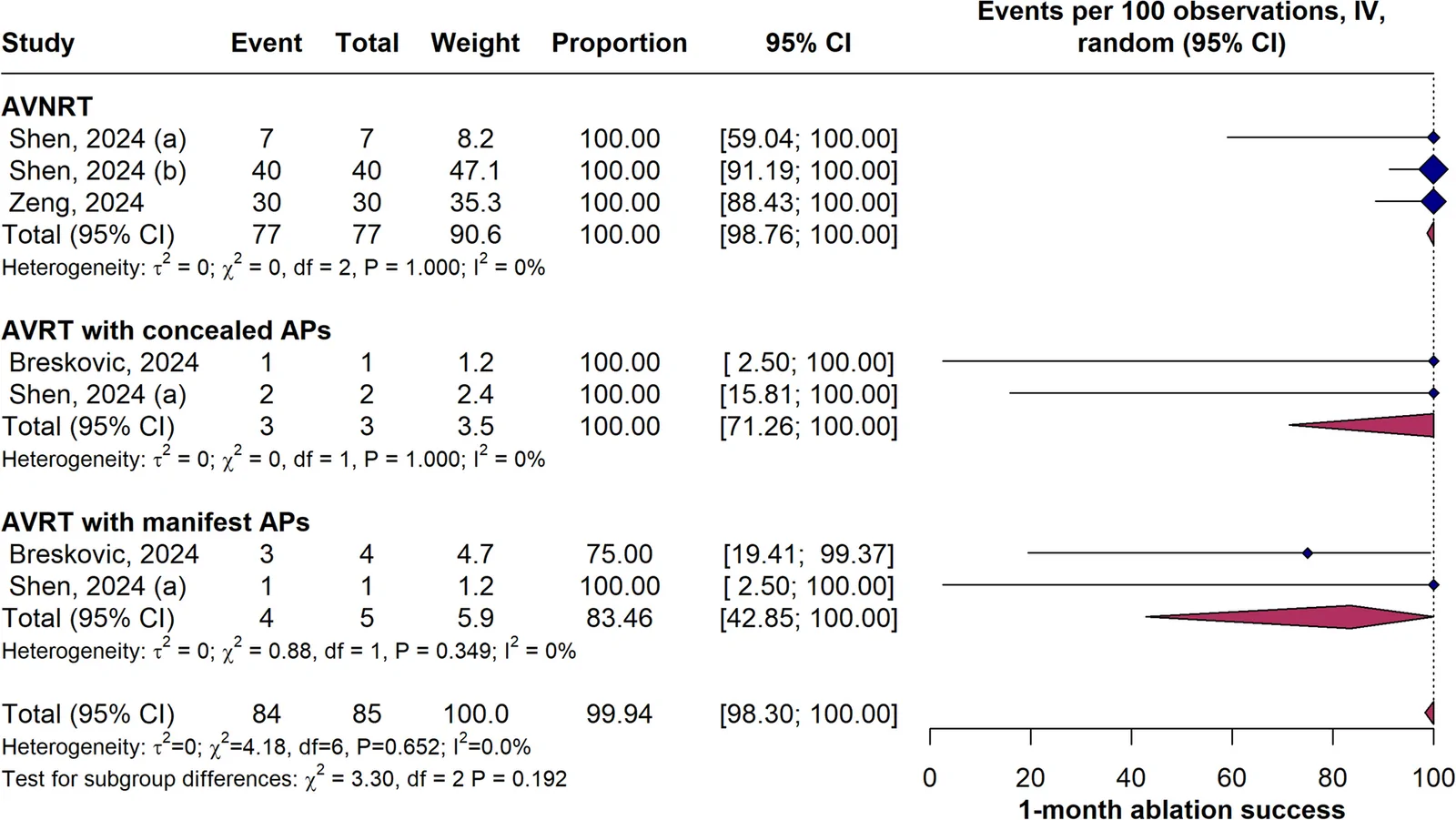

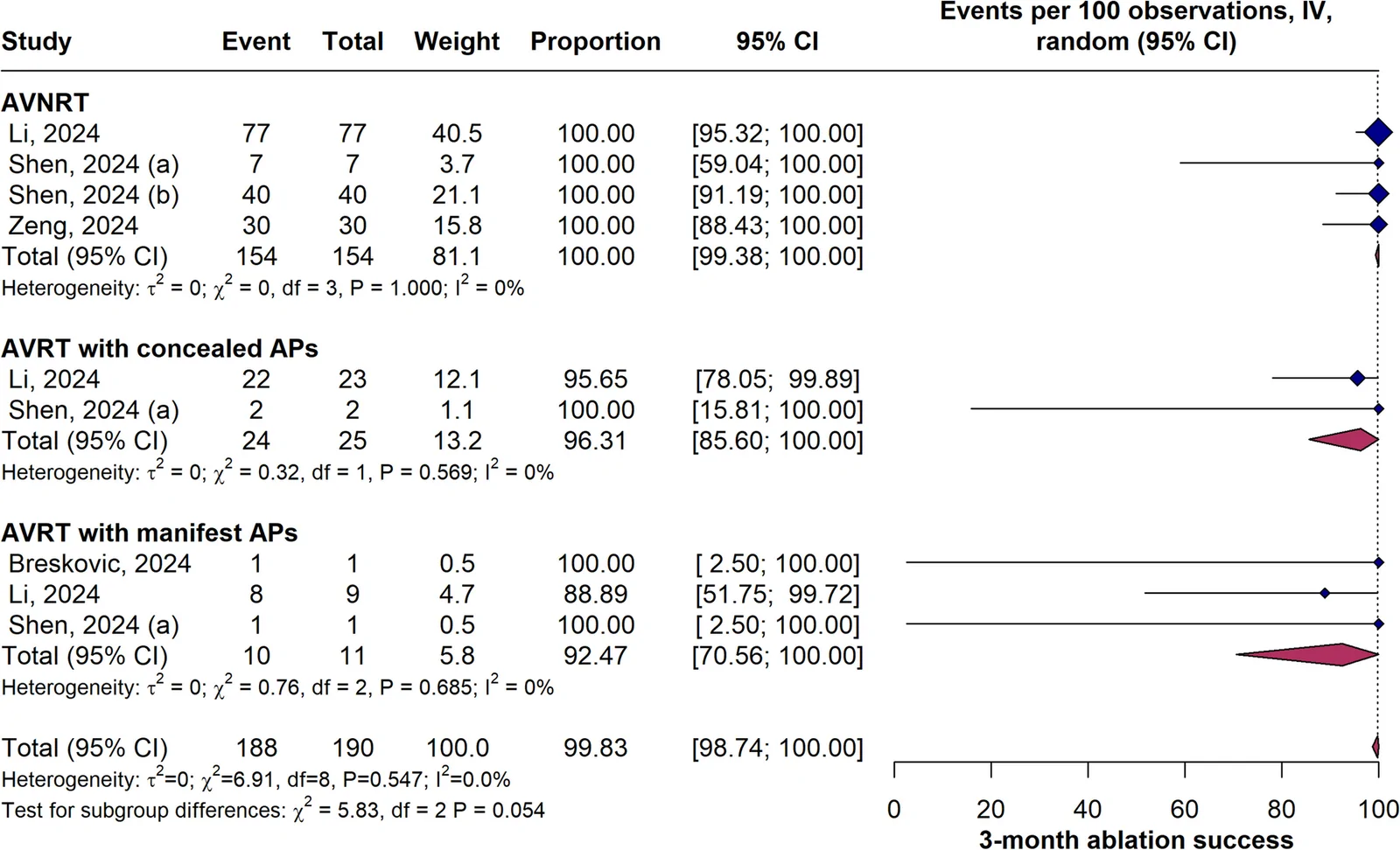

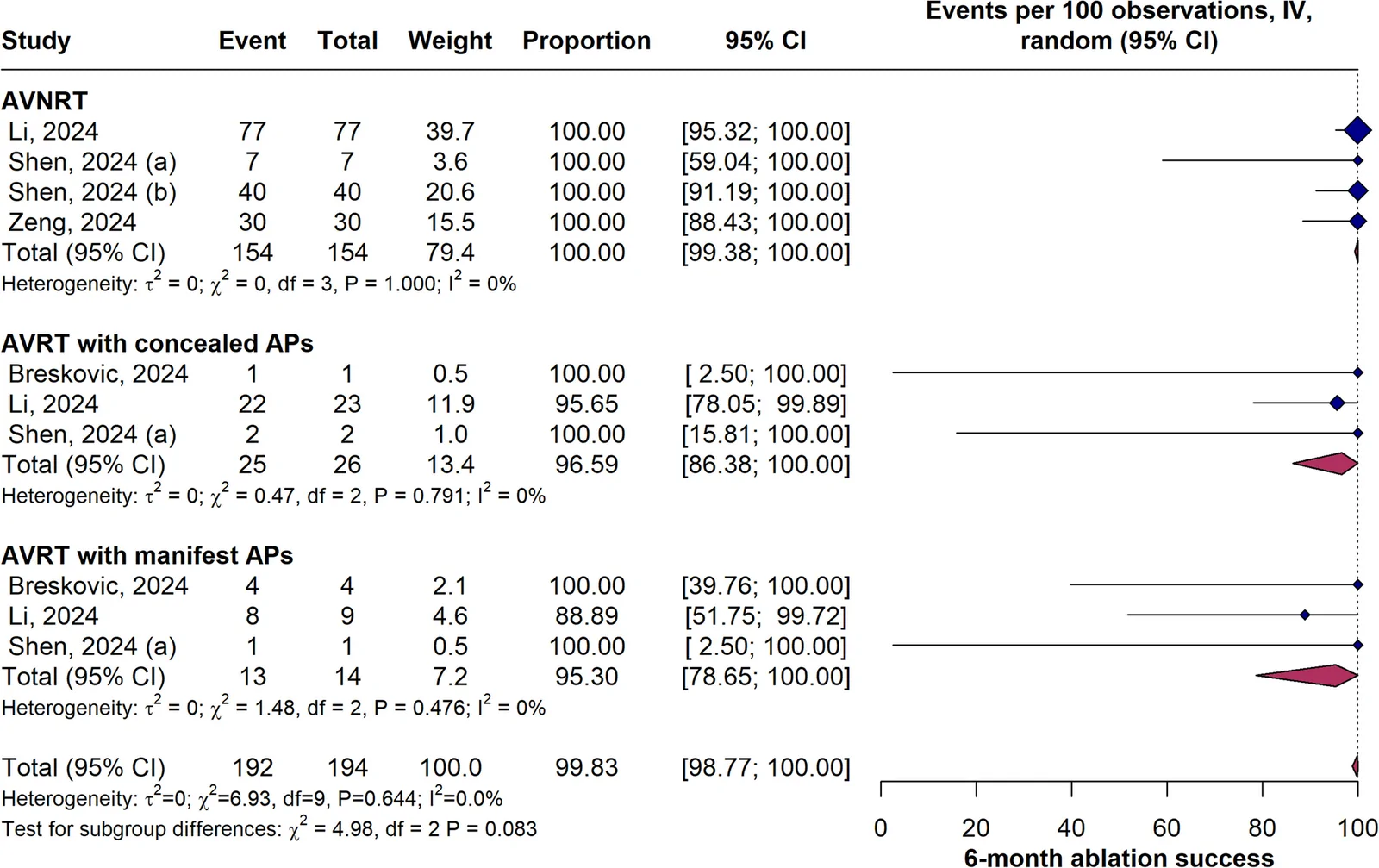

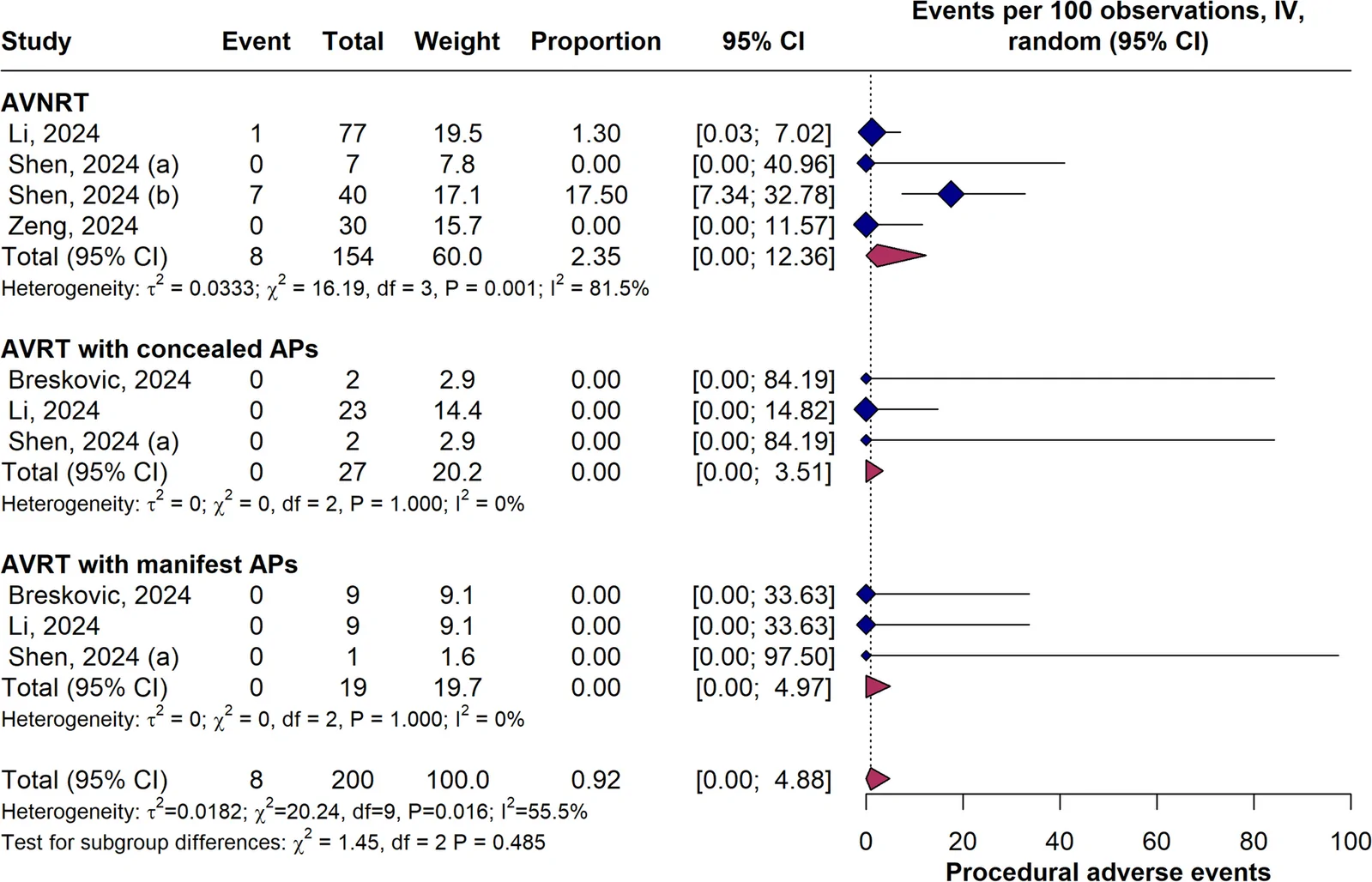

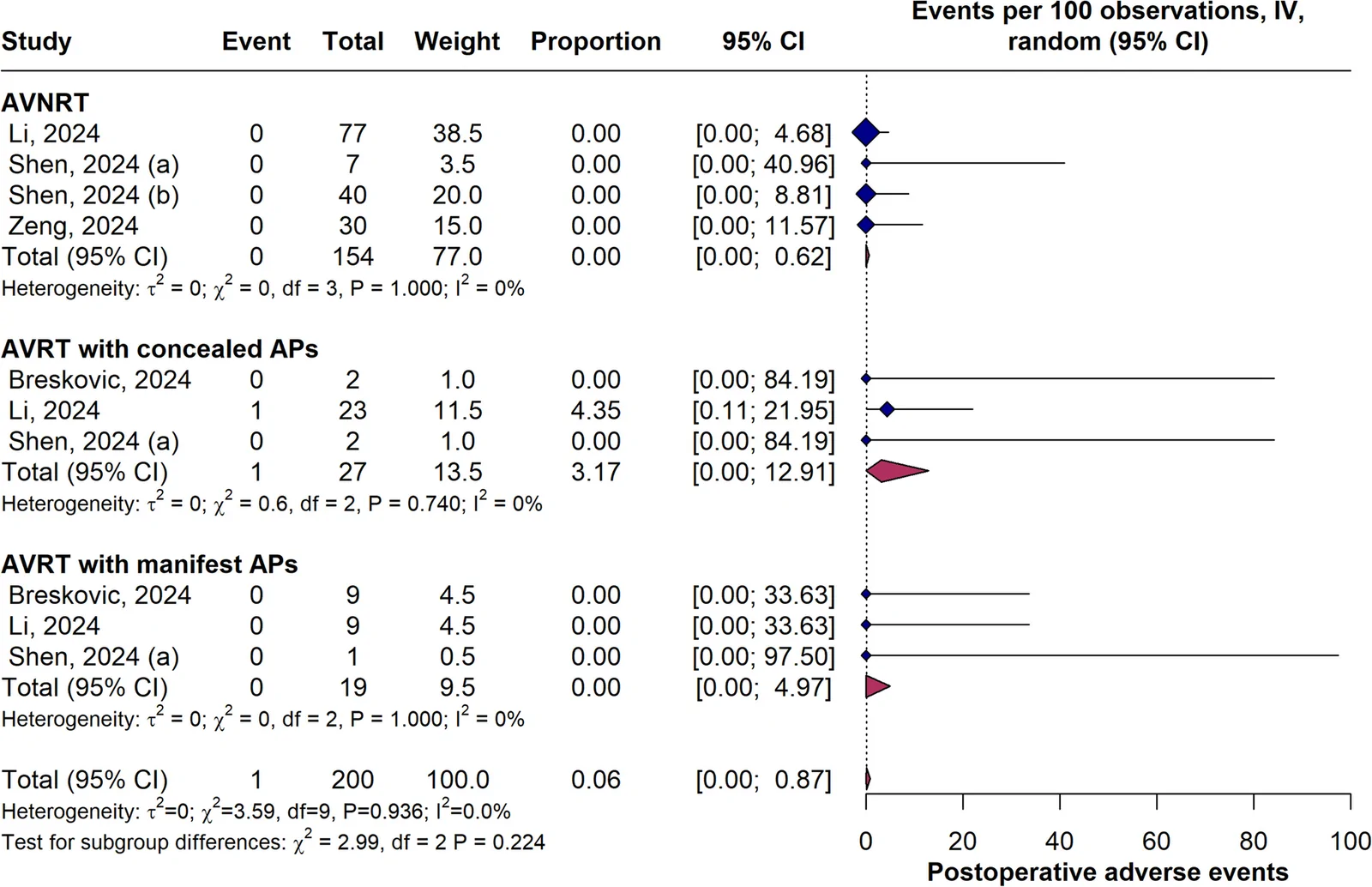
**a** Acute ablation success. The pooled acute ablation success rate for PFA was 99.98% (95% CI: 99.29–100; I^2^ = 0%). **b** 1-Month Ablation Success. The pooled ablation success rate at 1 month was 99.94% (95% CI: 98.30–100; I^2^ = 0%). **c** 3-Month Ablation Success. The pooled ablation success rate at 3 months was 99.83% (95% CI: 98.74–100; I^2^ = 0%). **d** 6-Month Ablation Success. The pooled ablation success rate at 6 months was 99.83% (95% CI: 98.77–100; I^2^ = 0%). **e** Incidence of Procedural Adverse Events. The pooled incidence of procedural AEs was 0.92% (95% CI: 0.00–4.88; I^2^ = 56%). **f** Incidence of Postoperative Adverse Events. The pooled incidence of postoperative AEs was 0.06% (95% CI: 0.00–0.87; I.^2^ = 0%)

The pooled acute procedural success rate for PFA was 99.98% (95% CI: 99.29–100; I^2^ = 0%, Fig. 2a) [2, 3, 9, 11, 12]. Success rates at 1, 3, and 6 months were 99.94% (95% CI: 98.30–100; I^2^ = 0%, Fig. 2b), 99.83% (95% CI: 98.74–100; I^2^ = 0%, Fig. 2c), and 99.83% (95% CI: 98.77–100; I^2^ = 0%, Fig. 2d), respectively. The overall incidence of procedural AEs was 0.92% (95% CI: 0.00–4.88; I^2^ = 56%, Fig. 2e). Postoperative AEs occurred in 0.06% of the cases (95% CI: 0.00–0.87; I^2^ = 0%, Fig. 2f).

As for procedural characteristics, the mean total ablation time was 5.36 s (95% CI: 1.56–9.16) [2, 3, 11, 12]. The average skin-to-skin procedure time was 86.15 min (95% CI: 73.20–99.10), with an average fluoroscopy time of 4.16 min (95% CI: 3.88–4.44). The average contact force was 8.56 g (95% CI: 6.41–10.72), and the mean distance from the closest ablation site to the bundle of His was 10.44 mm (95% CI: 6.99–13.90). Forest plots and subgroup analyses are available in the Supplementary Material (Figure S1).

### Subgroup analysis of selected populations

#### AVNRT

In the subgroup analysis of AVNRT, the pooled acute procedural success rate for PFA was 100% (95% CI: 99.38–100; I^2^ = 0%, Fig. 2a) [2, 3, 11, 12]. Success rate at 1 month was 100% (95% CI: 98.76–100; I^2^ = 0%, Fig. 2b), while 3 and 6 months were also 100% (95% CI: 99.38–100; I^2^ = 0%, Fig. 2c, d). The overall incidence of procedural AEs was 2.35% (95% CI: 0.00–12.36; I^2^ = 82%, Fig. 2e). In one study, six patients experienced transient third-degree AVB and one patient experienced transient second-degree AVB as procedural AE [2]. Another study reported one patient with transient first-degree AVB as a procedural AE [3]. Neither study required pacemaker implantation for AVB cases as they were transient and resolved within 24 h. Postoperative AEs occurred in 0% of cases (95% CI: 0.00–0.62; I^2^ = 0%, Fig. 2f).

#### AVRT with concealed APs

Upon an analysis of AVRT with concealed APs, the pooled acute procedural success rate for PFA was 100% (95% CI: 96.49–100; I^2^ = 0%, Fig. 2a) [3, 9, 11]. Success rate at 1 month was also 100% (95% CI: 71.26–100; I^2^ = 0%, Fig. 2b), while 3 and 6 months were 96.31% (95% CI: 85.60–100; I^2^ = 0%, Fig. 2c) and 96.59% (95% CI: 86.38–100; I^2^ = 0%, Fig. 2d), respectively. The overall incidence of procedural AEs was 0.00% (95% CI: 0.00–3.51; I^2^ = 0%, Fig. 2e). Postoperative AEs occurred in 3.17% of cases (95% CI: 0.00–12.91; I^2^ = 0%, Fig. 2f). Li et al. reported one postoperative AE with transient third-degree AVB [3]. Intravenous dexamethasone injection solved the condition.

#### AVRT with manifest APs

In the subgroup analysis of AVRT with manifest APs, the pooled acute procedural success rate for PFA was 97.47% (95% CI: 84.74–100; I^2^ = 9%, Fig. 2a) [3, 9, 11]. The success rate at 1 month was 83.46% (95% CI: 42.85–100; I^2^ = 0%, Fig. 2b), increasing to 92.47% at 3 months (95% CI: 70.56–100; I^2^ = 0%, Fig. 2c). At 6 months, the success rate was 95.30% (95% CI: 78.65–100; I^2^ = 0%, Fig. 2d). The overall incidence of procedural AEs was 0% (95% CI: 0.00–4.97; I^2^ = 0%, Fig. 2e), and postoperative AEs were also 0% (95% CI: 0.00–4.97; I^2^ = 0%, Fig. 2f).

### Sensitivity analysis

In the leave-one-out sensitivity analysis, no study showed dominance in terms of effect size (i.e., significantly altering the pooled proportion after removal) or heterogeneity (i.e., reducing I^2^ after removal).

We applied the Freeman-Tukey double arcsine transformation to all outcomes to assess the robustness of our statistical model. The results were consistent across all outcomes, including pooled proportions, heterogeneity, and study weight. Forest plots for these additional analyses are provided in the Supplementary Material (Figure S2).

### Quality assessment

All five studies were deemed to have a moderate risk of bias (Supplementary Material Figure S3). Inconsistent use of appropriate analysis methods to control important confounding factors was noted. Since comparison cohorts were not available, the balance of co-interventions could not be evaluated. Additionally, the awareness of outcome assessors regarding the chosen intervention contributed to a moderate risk of bias in outcome measurement.

## Discussion

In this systematic review and single-arm meta-analysis of five studies involving 202 patients, we evaluated the safety and feasibility of PFA for managing PSVT. The key findings from the pooled analyses were as follows: (1) the acute procedural success rate of PFA was exceptionally high at 99.98%, with success rates of 99.94%, 99.83%, and 99.83% observed at 1, 3, and 6 months, respectively; (2) the overall incidence of procedural AEs, including transient AVB, was 0.92%, whereas postoperative AEs were rare, occurring in only 0.06% of cases; (3) subgroup analyses of AVNRT showed a 100% success rate at all time points, with no postoperative AEs; and (4) for AVRT, the acute and 6-month success rates were 100% and 96.6% for concealed APs, and 97.5% and 95.3% for manifest APs, respectively, highlighting the potential of PFA as an effective and safe treatment option for these subtypes.

In the subgroup analysis, the lower success rate observed for AVRT with manifest accessory pathways should be interpreted with caution. This subgroup included only 19 patients across three studies, so even a single recurrence disproportionately affected the pooled estimate and widened the confidence intervals. Moreover, manifest pathways are often characterized by shorter effective refractory periods, making arrhythmia recurrence clinically easier to detect. Anatomically, fibers with anterograde conduction may extend deeper into the myocardial tissue, potentially increasing ablation complexity and reducing long-term procedural success. Larger studies are needed to clarify whether these findings reflect true biological differences or are primarily driven by small sample size effects.

PFA has been developed as a nonthermal, cardioselective ablative technique that permeabilizes cell membranes in arrhythmogenic foci, leading to cell death without significantly damaging the surrounding cardiac structures. In the setting of PSVT, PFA has the theoretical potential to provide effective and safe termination of arrhythmia, considering that atrioventricular conduction system cells contain fewer myofibrils and more connective tissue than typical cardiomyocytes, which increases their resistance to electroporation. These structural differences enhance the safety and precision of PFA, allowing it to target arrhythmogenic substrates while preserving critical conduction pathways [12]. Furthermore, fibrous sheaths, such as those surrounding the His bundle, help mitigate collateral damage during PFA, underscoring its effectiveness for treating PSVT [3, 11].

One of the advantages of PFA is PFA’s improved tissue selectivity [13]. Unlike RFA, which relies on thermal energy to create lesions, PFA could reduce the risk of steam pops, char formation, and thrombus formation by utilizing non-thermal irreversible electroporation [14]. Maintaining close catheter proximity remains essential for effective lesion formation [11].

Over the past years, PSVT ablation using RFA has demonstrated a favorable safety profile and high success rates. A meta-analysis of RFA ablation for PSVT found that RFA had a single-procedure success rate of 93.2% and a multiple-procedure success rate of 94.6%. Post-ablation arrhythmia occurred in 5.6% of cases, while 6.5% of patients required repeat ablation. The efficacy of RFA was slightly higher for AVNRT compared to accessory pathway tachycardias, with lower rates of recurrence and repeat ablation in AVNRT cases. The total proportion of patients with adverse events was 2.9%. The most commonly reported adverse events included AVB (1.4%) and need for pacemaker (0.65%) [15].

The study by Li et al. compared their results with a historical cohort of patients, matched for age and gender, who underwent RFA or cryoablation for PSVT. They found that PFA was associated with shorter procedural times, higher acute ablation success rates, lower short-term reablation rates, and fewer adverse events compared to traditional techniques. Furthermore, this study was the first to explore the combination of PFA and RFA, reporting that the hybrid approach was associated with higher efficacy and fewer adverse events compared to PFA alone [3]. The biophysics of PFA are not yet fully understood, but preliminary evidence suggests that dual-energy strategies or sequential RF and PFA applications may create more durable lesions[16]. Pre-heating tissue with RF could enhance PFA penetration and cell membrane disruption. Although these concepts remain speculative and require validation in larger studies, they offer a potential mechanistic explanation for the favorable outcomes observed with the hybrid approach.

To contextualize our findings, we summarized key differences between PFA and RFA based on data from previous studies [2, 3, 9, 11, 12, 15, 17, 18] (Table S2).

Breskovic et al. provided the first evidence of the effectiveness of PFA for AP ablation across multiple anatomical locations. APs in this cohort were classified as left-sided (lateral and posterior) and right-sided (free-wall and posteroseptal). Ablation of left-sided APs was primarily achieved via the coronary sinus (CS) in 5 of 6 patients. In one case of a left lateral with concealed AP, an initial attempt using the CS approach was ineffective, necessitating a switch to a retrograde transaortic route, which successfully terminated conduction. Notably, no acute complications were observed during these procedures. Right-sided APs were also effectively treated without AEs, demonstrating the safety and efficacy of PFA across various anatomical sites. Notably, this is the only study with a follow-up period exceeding six months, showing no recurrence of PSVT or AEs, providing key insights into PFA's durability. Additionally, it was the first to include pediatric patients, demonstrating a 100% success rate both intraoperatively and during follow-up [9].

While the procedural safety profile PFA is promising, certain challenges remain. Shen et al. reported that seven patients (17.5%) experienced transient AVB, and eight patients (20%) had mild skeletal muscle contractions. In three of these cases (42.9%), AVB was linked to abrupt catheter movement caused by slight body shifts or deep breaths, particularly when the distance between the ablation site and the largest His activation was shorter, leading to a higher incidence of transient AVB [2]. Zeng et al. found no cases of AVB, even when the PFA catheter was within 2 mm of the closest ablation site to the His bundle [12]. This discrepancy may reflect anatomical variability, which can influence procedural outcomes. Differences in the composition of fast and slow pathways are likely to explain why some patients develop AVB while others do not. The slow pathway, composed mainly of cardiomyocytes, is more susceptible to PFA, whereas the fast pathway, consisting of conduction bundles, is less affected [2, 12]. When compared to traditional RF ablation, where transient AVB is typically observed in only 1–2% of cases [19], the AVB rate reported in Shen et al.'s study stands out significantly. The higher rate of transient AVB reported with PFA, albeit from a single study, raises concerns about the potential for unintended conduction system damage, particularly in cases where catheter movement or proximity to critical conduction structures is a factor. However, it is important to note that in all cases with transient AVB, no patients required permanent pacemaker implantation, as the conduction disturbances resolved spontaneously [2]. Another study reported a case of a patient who experienced a transient third-degree AVB. The patient had two APs: one located in the right anteroseptal region and the other in the right lateral region. Consequently, the number of ablation procedures and the cumulative ablation time were significantly higher, potentially leading to myocardial edema. Furthermore, one of the ablation sites was situated near the His bundle, which may have played a role in the development of the AVB. The condition was resolved with the administration of intravenous dexamethasone [3]. AV block is considered an exceedingly rare complication in the field of electrophysiology; however, its occurrence in the context of SVT ablation, where the majority of patients are very young, is particularly concerning. PFA may offer a theoretical safety advantage over RF in cases such as mid-septal accessory pathways located in close proximity to the conduction system, since electroporation can be reversible depending on pulse parameters. In this setting, lesion delivery could potentially be aborted if conduction disturbances were detected, similar to strategies previously adopted with cryoablation, although the cost-effectiveness of such an approach remains uncertain. Furthermore, procedural factors such as performing ablations under general anesthesia to reduce patient movement may be especially relevant for PFA, as muscle capture and even phrenic nerve involvement are commonly observed during energy delivery.

Nonetheless, large randomized trials are warranted to ascertain the comparative efficacy and safety of PFA versus traditional thermal techniques as well as the long-term outcomes of PFA in patients with PSVT. Even so, our analysis is the most powerful and largest to evaluate the role of this promising technique in this scenario, providing valuable insights in this relatively unexplored field.

This meta-analysis offers valuable insights into the emerging role of PFA for PSVT; however, several important limitations should be considered when interpreting the results. All included studies were single-arm, non-randomized trials without control groups, limiting the ability to draw comparative conclusions regarding the efficacy and safety of PFA versus standard ablation techniques such as radiofrequency or cryoablation. Moreover, most studies originated from China and employed novel catheters not yet widely adopted in clinical practice, which may limit the generalizability of our findings to other populations and settings. Nonetheless, we pooled all available eligible studies to provide the most comprehensive synthesis to date, aiming to increase statistical power and generalizability. The total sample size was modest (*n* = 202), and most studies reported follow-up of only up to six months, which restricted evaluation of long-term durability, arrhythmia recurrence, and delayed complications. Although one study extended follow-up beyond six months, the dataset overall did not support time-to-event analyses or survival modeling. Moderate heterogeneity (I^2^ = 56%) was observed in procedural adverse events. Because of the limited number of included studies and lack of covariate-level data, meta-regression was not feasible. Instead, we conducted predefined subgroup analyses by arrhythmia subtype (AVNRT and AVRT) to partially account for clinical heterogeneity and assess the consistency of results. The overall risk of bias was rated as moderate across all studies using the ROBINS-I tool. Contributing factors included lack of adjustment for potential confounders, selection bias related to the inclusion of patients with structurally normal hearts, non-standardized ablation protocols, and variability in definitions of procedural success and adverse events. Although these factors could have influenced pooled estimates, the limited number of studies precluded formal modeling of bias impact. We did not conduct a formal assessment of publication bias (e.g., funnel plot or Egger’s test), as these methods have limited interpretability in meta-analyses of proportions with a small number of included studies. Additionally, the inclusion of only five studies precluded reliable visual or statistical detection of asymmetry. However, to mitigate potential reporting bias, we conducted a comprehensive search without language or date restrictions and reviewed clinical trial registries and reference lists of included studies. While stratified analyses were performed, the primary pooled estimates combined AVNRT and AVRT cases, which differ in pathophysiology and procedural objectives. This may have obscured relevant differences in procedural outcomes. Additionally, the novelty of PFA in this clinical setting meant that ablation parameters, catheter types, and procedural endpoints were not standardized across studies, introducing further heterogeneity. We acknowledge that two studies, particularly the largest one with 111 patients, contributed most of the weight to the pooled analysis, which may have influenced the summary estimates; however, pooling all available evidence still provides the most comprehensive assessment of PFA for PSVT to date.

Despite these limitations, we strengthened the methodological rigor of our work through protocol pre-registration, sensitivity analyses and stratified subgroup assessments. These measures enhance the reliability of our findings and underscore the need for future multicenter, randomized trials with standardized methodology and extended follow-up to more clearly define the role of PFA in the management of PSVT.

## Conclusion

In this meta-analysis of proportions, PFA has shown significant promise as a non-thermal ablation modality for treating PSVT, particularly AVNRT. The pooled acute ablation success rate was 99.98%, with sustained success observed at 1, 3, and 6 months. Additionally, PFA exhibited a favorable safety profile characterized by minimal AEs and preservation of surrounding tissue. Randomized controlled trials are warranted to establish the long-term efficacy and safety of PFA in this setting.

## Supplementary Information

Below is the link to the electronic supplementary material.

Supplementary file1 (DOCX 3158 KB)

## Data Availability

All data used in this study are from published sources and are available in the references cited.

## Abbreviations

AE: Adverse event
AP: Accessory pathway
AVB: Atrioventricular block
AVNRT: Atrioventricular nodal reentrant tachycardia
AVRT: Atrioventricular reentrant tachycardia
CI: Confidence interval
PFA: Pulsed field ablation
PSVT: Paroxysmal supraventricular tachycardia
RFA: Radiofrequency ablation

## Declarations

### Registry and the registration No. of the study/trial

PROSPERO number: CRD42024621050.
