# Supplementary material for: Pulsed field ablation for paroxysmal supraventricular tachycardia: a systematic review and single-arm meta-analysis
Source: J Interv Card Electrophysiol. 2025 Nov 21;69(7):1997–2009. doi: 10.1007/s10840-025-02166-w (PMC13687147; doi:10.1007/s10840-025-02166-w)
Supplement: Supplementary file 1 [file 10840_2025_2166_MOESM1_ESM.docx]

**SUPPLEMENTARY MATERIAL**

**Pulsed Field Ablation for Paroxysmal Supraventricular Tachycardia: A Systematic Review and Single-Arm Meta-Analysis**

**Journal of Interventional Cardiac Electrophysiology**

Giang Son Arrighini ^1^, Mrunalini Dandamudi, MD ^2^, Vinh Ho Quang Tri ^3^, David Downes ^4^, Nicole Felix, MD ^5^, Vanio do Livramento Junior Antunes ^6^, Guilherme Dagostin de Carvalho, MD, MHSc ^7^, Iuri Ferreira Felix, MD ^8^, Victoria Zecchin Ferrara ^9^, Hemank Walia, MD ^10^, Igor Diemberger MD^11^, Gabriel Odozynski, MD^12^, Andre d’Avila, MD^13^, Juliana Giorgi, MD ^14,15^

1- Faculty of Medicine and Surgery, University of Bologna, Bologna, Italy.
2- Montefiore Einstein Hospital, Bronx, New York, United States.
3- Faculty of Medicine, University of Debrecen, Debrecen, Hungary.
4- Department of Rural Medicine, The University of New England, Armidale, NSW, Australia.
5- Federal University of Campina Grande, Campina Grande, PB, Brazil.
6- Federal University of Health Sciences of Porto Alegre, Porto Alegre, RS, Brazil.
7- Dante Pazzanese Institute of Cardiology, Department of Cardiac Arrhythmias and Electrophysiology, São Paulo, Brazil.
8- Department of Medicine, Mayo Clinic, Rochester, Minnesota, United States.
9- Faculty of Medicine and Surgery, University of Padua, Padua, Italy.
10- Advocate Illinois Masonic Medical Center, Chicago, Illinois, United States.
11- Department of Cardio-Thoraco-Vascular Sciences, Cardiology Unit, IRCCS Azienda Ospedaliero-Universitaria di Bologna, Bologna, Italy.
12- Harvard-Thorndike Electrophysiology Institute, Beth Israel Deaconess Medical Center, Harvard Medical School, Boston, Massachusetts, United States.
13- Harvard-Thorndike Electrophysiology Institute, Beth Israel Deaconess Medical Center, Harvard Medical School, Boston, Massachusetts, United States.
14- Hospital Sírio-Libanês, São Paulo, SP, Brazil.
15- Hospital Albert Einstein, São Paulo, SP, Brazil.

**Corresponding author:** Giang Son Arrighini

**Affiliation:** Faculty of Medicine and Surgery, University of Bologna, Bologna, Italy

**E-mail address:** [giangson.arrighini@studio.unibo.it](mailto:giangson.arrighini@studio.unibo.it)

**ORCID:** 0009-0009-6830-5453

*Table S1: procedural characteristics*

*Figure S1: pooled analysis of procedural characteristics*

Figure S1b. Distance from the closest ablation site to His bundle (millimeters)

Figure S1a. Average contact force (grams)

Figure S1d. Fluoroscopy time (minutes)

Figure S1c. Cumulative effective PFA application time (milliseconds)

Figure S1f. Skin-to-skin procedure time (minutes)

Figure S1e. Number of PFA applications

Figure S1g. Total ablation time (seconds)

*Figure S2: leave-one-out sensitivity analysis*

Figura S2a. Acute ablation success

Figure S2c. 3-month ablation success

Figure S2b. 1-month ablation success

Figure S2d. 6-month ablation success

Figure S2d. Procedural adverse events

Figure S2e. Postoperative adverse events

Table S2: PFA vs RFA: Key differences

| **Domain** | **Metric / Definition** | **PFA (pooled, this meta-analysis)** | **RFA (benchmark from SVT literature)** |
| --- | --- | --- | --- |
| **Efficacy** | **Acute procedural success** | 99.98% (95% CI 99.29–100; I²=0%) | ≈95–98% |
|  | **Success at 1 month** | 99.94% (95% CI 98.30–100; I²=0%) | ≈94–97% |
|  | **Success at 3 months** | 99.83% (95% CI 98.74–100; I²=0%) | ≈92–96% |
|  | **Success at 6 months** | 99.83% (95% CI 98.77–100; I²=0%) | ≈90–95% |
| **Safety** | **Procedural adverse events (composite)** | 0.92% (95% CI 0.00–4.88; I²=56%) | ~1–3% major complications |
|  | **Post-operative adverse events** | 0.06% (95% CI 0.00–0.87; I²=0%) | (rare; typically included in composite outcome) |
|  | **Procedural mortality** | Not reported in our pooled analysis | <0.1% |
| **Procedural Characteristics** | **Total ablation time** | 5.36 s (95% CI 1.56–9.16) | Longer; point-by-point lesion creation (no single pooled number consistently reported) |
|  | **Skin-to-skin procedure time** | 87.77 min (95% CI 77.74–97.79) | Typically longer; varies by substrate/operator |
|  | **Fluoroscopy time** | 6.98 min (95% CI 2.06–11.90) | Variable; often higher in conventional workflows |
|  | **Mean contact force** | 8.56 g (95% CI 6.41–10.72) | Not applicable (RFA efficacy depends on contact force, but no single benchmark) |
|  | **Distance to His bundle (closest lesion)** | 10.44 mm (95% CI 6.99–13.90) | Not routinely summarized across studies |
| **Mechanism & Risk Profile** | **Mechanism of action** | Non-thermal, irreversible electroporation | Thermal resistive heating |
|  | **Tissue selectivity** | Higher myocyte selectivity; spares adjacent structures | Non-selective; risk to adjacent tissues |
|  | **Thermal injury risk** | Minimal | Present (e.g., steam pops, char, collateral injury) |
|  | **Nerve/vascular effects** | Lower risk of permanent nerve injury; possible hemolysis with high-field pulses; larger sheath may increase vascular-access issues | Phrenic/other nerve injury possible; thrombus/char risk |
|  | **Clinical adoption** | Newer, rapidly expanding | Long-standing standard of care |

Figure S3. Risk of Bias Assessment. Risk of bias analysis of included studies using the ROBINS- I tool.
